# Genome-Wide Analysis of Allele-Specific Expression Patterns in Seventeen Tissues of Korean Cattle (Hanwoo)

**DOI:** 10.3390/ani9100727

**Published:** 2019-09-26

**Authors:** Kyu-Sang Lim, Sun-Sik Chang, Bong-Hwan Choi, Seung-Hwan Lee, Kyung-Tai Lee, Han-Ha Chai, Jong-Eun Park, Woncheoul Park, Dajeong Lim

**Affiliations:** 1 Department of Animal Science, Iowa State University, Ames, IA 50011, USA; kyusang0912@gmail.com; 2 Hanwoo Research Institute, National Institute of Animal Science, Rural Development Administration, Pyeongchang 25340, Korea; jangsc@korea.kr; 3 Animal Genomics and Bioinformatics Division, National Institute of Animal Science, Rural Development Administration, Wanju 55365, Korea; bhchoi@korea.kr (B.-H.C.); leekt@korea.kr (K.-T.L.); hanha@korea.kr (H.-H.C.); jepark0105@korea.kr (J.-E.P.); wcpark1982@korea.kr (W.P.); 4 Division of Animal and Dairy Science, Chungnam National University, Daejeon 34134, Korea; slee46@cnu.ac.kr

**Keywords:** allele-specific expression, RNA-Seq, Hanwoo

## Abstract

**Simple Summary:**

Allele-specific expression (ASE) is the biased allelic expression of genetic variants within the gene. Recently, the next-generation sequencing (NGS) technologies allowed us to detect ASE genes at a transcriptome-wide level. It is essential for the understanding of animal development, cellular programming, and the effect on their complexity because ASE shows developmental, tissue, or species-specific patterns. However, these aspects of ASE still have not been annotated well in farm animals and most studies were conducted mainly at the fetal stages. Hence, the current study focuses on detecting ASE genes in 17 tissues in adult cattle. In particular, we analyzed the monoallelic expression (MAE), which is a subtype of ASE where only one of two alleles are expressed.

**Abstract:**

The functional hemizygosity could be caused by the MAE of a given gene and it can be one of the sources to affect the phenotypic variation in cattle. We aimed to identify MAE genes across the transcriptome in Korean cattle (Hanwoo). For three Hanwoo family trios, the transcriptome data of 17 tissues were generated in three offspring. Sixty-two MAE genes had a monoallelic expression in at least one tissue. Comparing genotypes among each family trio, the preferred alleles of 18 genes were identified (maternal expression, n = 9; paternal expression, n = 9). The MAE genes are involved in gene regulation, metabolic processes, and immune responses, and in particular, six genes encode transcription factors (*FOXD2*, *FOXM1*, *HTATSF1*, *SCRT1*, *NKX6*-*2*, and *UBN1*) with tissue-specific expression. In this study, we report genome-wide MAE genes in seventeen tissues of adult cattle. These results could help to elucidate epigenetic effects on phenotypic variation in Hanwoo.

## 1. Introduction

Allele-specific expression (ASE) is a differential abundance of the allelic copies of a transcript having unequal expression if an individual has a heterozygous site in the coding sequence of the gene. This is caused by *cis*-regulatory elements, such as *cis*-acting DNA sequence variants, epigenetic marks, and post-transcriptional modifications. Various studies performed on mice and humans show that these variations affect the level of expression as showing allele-specific expression or allelic imbalance. Several ASE genes are identified in livestock, such as Marek’s disease of chicken [1,2], uterine milk protein of dairy cattle [3], or spermatogenesis or lipid metabolism of pigs [4]. Thus, identifying ASE is important to explain the gap between genotypes and phenotypic variations in economic traits. In particular, some ASEs are caused by genomic imprinting depending on the parental origin which has a monoallelic expression (MAE) pattern. Recently, it has become possible to analyze MAE at the whole transcriptome level using RNA sequencing (RNA-seq) and whole-genome resequencing (Re-seq), and these approaches were applied to identify monoallelic expression patterns of MAE genes in humans placenta [5], mice brain [6], chicken embryos [7], and bovine conceptus [8]. These epigenetic phenomena refer to alterations of gene expression without a change of DNA sequence [9,10,11]. In particular, studies of MAE patterns in livestock are relatively limited compared to other species such as humans or mice, and not all MAE genes found in humans and mice show MAE patterns in other animals. In addition, MAE including genomic imprinting has developmental stage-, species, or tissue-specific patterns. For examples, *IPL* gene has fetal-specific expression such as MAE gene that is monoallelically expressed in all tissues in newborn pigs but biallelically expressed in those tissues from adult pigs [12]. In cattle, *IGF2* gene shows paternal expression in fetal tissues [13,14] but the maternal alleles are expressed in the adult tissues, such as heart and liver [14].

Here, we investigate ASE in 17 different tissues from each offspring in three Hanwoo family trios and focus on MAE among ASE based on Re-Seq and RNA-Seq. We also found that several MAE showed tissue-specific patterns and bias towards the expression of the paternal or maternal allele.

## 2. Materials and Methods

### 2.1. Ethics Statement

This study was approved by the local committees and the research ethics committees of the National Institute of Animal Science, Republic of Korea. The experimental protocols were approved by the Committee on the Ethics of Animal Experiments of the National Institute of Animal Science (Permit Number: NIAS2015-776).

### 2.2. Sample Collection

To identify MAE in Hanwoo, we used a single-nucleotide polymorphism (SNP)-based approach for whole-genome and transcriptome data [5,7]. In previous studies on the MAE, including genomic imprinting of animal models, the reciprocal crossing of different breeds or lines was applied to increase heterozygosity in the tested animals [7]. However, Hanwoo is an indigenous cattle breed and has been maintained as a purebred (single line) for meat type in South Korea. Instead of the reciprocal crossing method, we used three family trios to reduce false-positive information.

A total of seven animals from three family trios (sire, dam, and offspring) were used from the Hanwoo Research Institute, National Institute of Animal Science, Republic of Korea. Three dams were crossed with the same sire, so three male offspring were half-siblings. The peripheral blood samples from all animals were collected for DNA extraction. Offspring were slaughtered at 37 months of age, and the 17 tissues, including blood, were immediately sampled for RNA extraction. The samples and their abbreviations in this study are as follows: pituitary gland, PG; hypothalamus, HH; spinal cord, SC; lymph node, LY; spleen, SP; liver, LV; large intestine, LI; small intestine, SI; heart, HE; loin muscle, LM; femoral muscle, FM; kidney, KD; adrenal gland, AG; blood, BL; lung, LU; cerebrum, CR; cerebellum, CL.

Genomic DNA and total RNA were extracted using the QIAamp DNA Blood Maxi Kit (Qiagen, Gaithersburg, MD, USA) and TRIzol reagent (Life Technologies Corp., Grand Island, NY, USA), in accordance with the manufacturers’ instructions, respectively. Agarose gel electrophoresis and a Nanodrop spectrophotometer (Nanodrop Technologies Inc., Wilmington, DE, USA) were employed for quality controls of DNA and RNA. The RNA quality was analyzed by the Agilent 2100 Bioanalyzer (Agilent Technologies, Palo Alto, CA, USA) and the RNA samples with the RNA integrity number 7.0 or above were used in RNA-sequencing.

### 2.3. Whole-Denome Resequencing and RNA-Sequencing

For whole-genome resequencing, indexed shotgun paired-end libraries were generated using the TruSeq Nano DNA Library Prep Kit (Illumina, San Diego, CA, USA), in accordance with the manufacturer’s instructions. The size-selected libraries (550 to 650 bp) were analyzed using an Agilent 2100 Bioanalyzer (Agilent Technologies, Palo Alto, CA, USA), and checked for adapter contamination. Next, the libraries were sequenced by an Illumina HiSeq 2500 (2 × 125-bp paired-end sequences) sequencer. Duplicated reads were removed and alignment information was sorted using Picard tools (http://picard.sourceforge.net) and Samtools [15]. The raw reads were mapped to the *Bos taurus* reference genome (UMD 3.1) using Bowtie2 with default option settings [16].

For RNA-seq, cDNA was synthesized by SuperScript III Reverse Transcriptase (Life Technologies Corp., Grand Island, NY, USA), in accordance with the manufacturer’s instructions. Then, the Illumina-compatible libraries were constructed using a TruSeq RNA library preparation kit (Illumina, San Diego, CA, USA), following the manufacturer’s instructions. Sequencing was conducted with paired-end 101 bp using the HiSeq 2000 platform (Illumina, San Diego, CA, USA). Quality control of fastq sequence reads was performed using FastQC software [17]. Transcripts were then assembled in Cuffdiff program, and FPKM (Fragments Per Kilobase of transcript pre-Million mapped reads) values for gene expression levels were obtained.

### 2.4. Identification of Imprinted Genes

SNP calling was conducted using the genome analysis toolkit (GATK, version 3.6) HaplotypeCaller with standard settings, and SNP annotation using SnpEff with the cattle reference genome (UMD3.1.78) was performed to capture only exonic SNPs. These exonic SNPs were then filtered using the following criteria: QD < 2.0, FS > 60.0, MQ < 40.0, MQRankSum < −12.5, ReadPosRankSum < −8.0, GQ < 20, and DP < 7. Finally, heterozygote SNPs in all offspring after removing family trio genotype error were gathered as informative SNPs for further analysis.

To determine the presence of MAE, the following steps were performed for each tissue (Figure S1): (i) identification of SNPs with allele-specific expression (monoallelic expression) in all offspring, (ii) screening for candidate imprinted genes with at least one allele-specific expressed SNP, (iii) calculating the ratio of allele-specific expressed SNPs among all SNPs (ASE%) in each candidate gene, and (iv) classifying the genes having ASE% of above 75% as MAE, as described by the previous study [5].

### 2.5. Functional Annotation Analysis

The DAVID database was used for functional enrichment analysis. Ensembl gene IDs were submitted to DAVID and *Bos taurus* was selected as a species. Gene ontology for GOTERM_BP_FAT was used to obtain the required data.

### 2.6. Experimental Validation of Monoallelically Expressed Genes Using Direct Sequencing

To validate the monoallelic expression of the tested genes identified in this study, polymerase chain reaction (PCR) and direct sequencing were performed using DNAs and synthesized cDNAs with specific primer sets (Table S1). PCR products were purified and Sanger sequencing was performed using the BigDye Terminator Cycle Sequencing Ready Reaction Kit (ver. 3.0; Life Technologies Corp., Grand Island, NY, USA) and an ABI PRISM^®^ 3730 Genetic Analyzer (Life Technologies Corp., Grand Island, NY, USA). The sequence analysis for SNP loci was performed using SeqMan software (DNASTAR).

## 3. Results

### 3.1. Identification of Heterozygous SNPs Based on NGS Sequencing Data

In this study, we generated approximately 2.26 billion paired-end reads using re-sequencing for all seven animals, and an average of 98.63% of reads were mapped to the bovine reference sequence assembly (Table S2). In the variant calling, 9,749,237 SNPs were obtained from Re-seq (Figure S2). Among them, only 136,496 SNPs (1.4% of total called SNPs) were located in the exonic regions. The heterozygote genotypes are essential to determine whether a given gene shows MAE, and each offspring had heterozygous genotypes in 39,116, 32,389, and 31,855 SNP loci (Figure S3). We kept the informative SNPs (n = 4470) which were heterozygous in all offspring, and these were included within 1681 genes.

For RNA-Seq, an average of 85.01% of reads were mapped to the reference genome (Table S3). Transcriptome-derived genotypes which had the expressed alleles at each locus were obtained by HaplotypeCaller in GATK. In informative SNP locus, the MAE of each SNP was defined if transcriptome-derived genotypes in all offspring were homozygous, which indicated expression of one allele on RNA level. As shown in Table 1, tested SNPs were successfully genotyped from RNA-sequencing data in all offspring. To avoid false positive error in the test for MAE, strict criteria were applied in the further analysis. Only SNPs genotyped in RNA-seq of all offspring in each tissue were tested. Therefore, the numbers of tested SNPs per tissue varied within the range from 508 to 1131 (from 340 to 699 in the tested genes) because of tissue-specific expression and/or missing genotypes. In addition, the criterion for allele-specific expression percent (ASE%), which was the proportion of imprinted SNPs within all SNPs among the tested genes, of 75% was applied for the discrimination of imprinted genes, as explained in the previous study [7]. 

### 3.2. Identification of Monoallelic Expression in Korean Cattle (Hanwoo)

The numbers of imprinted SNPs and genes per tissue are shown in Table 1. Lymph node and kidney showed the highest number of imprinted genes (n = 32), while spinal cord showed the lowest (n = 18). However, the proportions of imprinted genes among all tested genes were similar in all tissues. Approximately 5% of tested genes showed MAE in each tissue.

**Figure 1:**
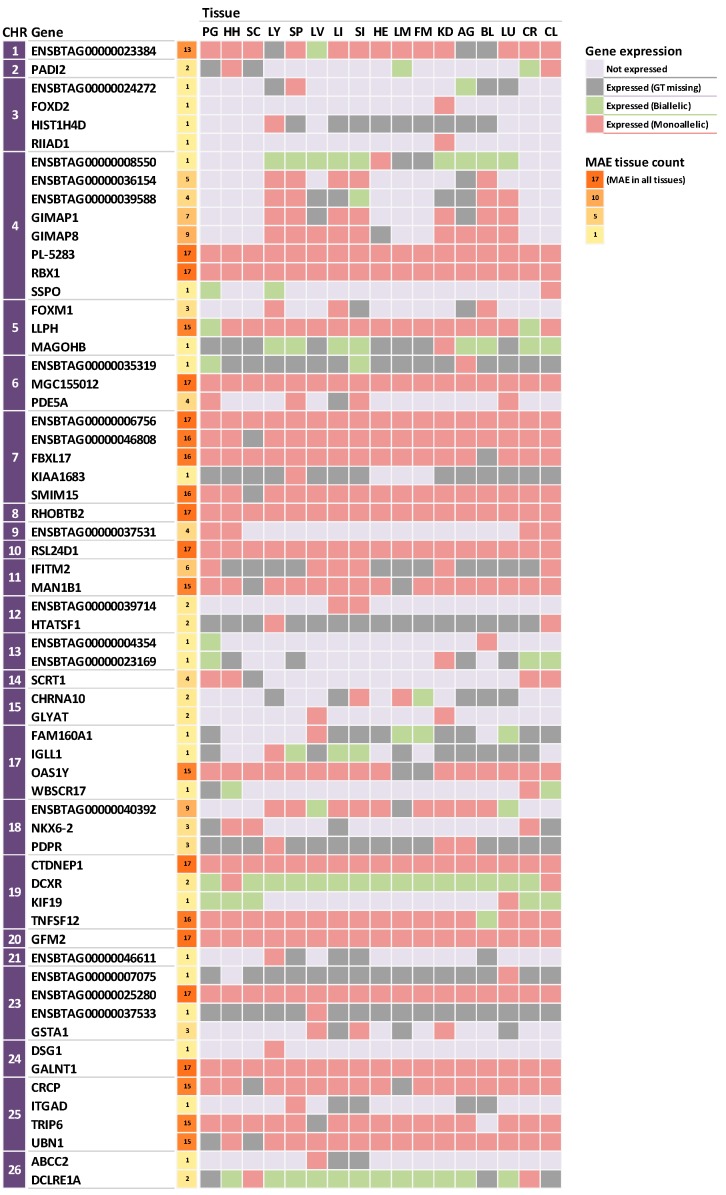
Monoallelic expression (MAE) of 62 genes in 17 tissues. The expression levels below FPKM 1 were considered as absence of expression and are represented by light grey squares. FPKM, fragments per Kilobase of exon per million fragments mapped; CHR, chromosome.

Among the MAE genes identified in this study, 10 genes (*CTDNEP1*, *ENSBTAG00000006756*, *ENSBTAG00000025280*, *GALNT1*, *GFM2*, *MGC155012*, *PL-5283*, *RBX1*, *RHOBTB2*, *RSL24D1*) showed MAE in all tested tissues (Figure 1). In addition, there were tissue specific expressed genes, such as *FOXD2*, *RIIAD1* and *DSG1*. The former two genes are expressed in the kidney and the latter in only the lymph node. MAE were also observed in 20 genes, such as *PADI2* and *SSPO*, which showed that biallelic and monoallelic expressions are tissue specific.

Furthermore, the genes for which the parental origins of the alleles could be defined definitively by comparing with the parental genotypes were as follows: nine genes for which the maternal allele was expressed (*DCLRE1A*, *DSG1*, *ENSBTAG00000037533*, *ENSBTAG00000046611*, *ENSBTAG00000046808*, *GLYAT*, *HTATSF1*, *MGC155012*, *SSPO*) and nine genes for which the paternal allele was expressed (*CTDNEP1*, *ENSBTAG00000039714*, *GFM2*, *GSTA1*, *IFITM2*, *PL-5283*, *RIIAD1*, *RSL24D1*, *SCRT1*).

### 3.3. Tissue-Specific Expression Patterns of Monoallelically Expressed Genes

**Figure 2:**
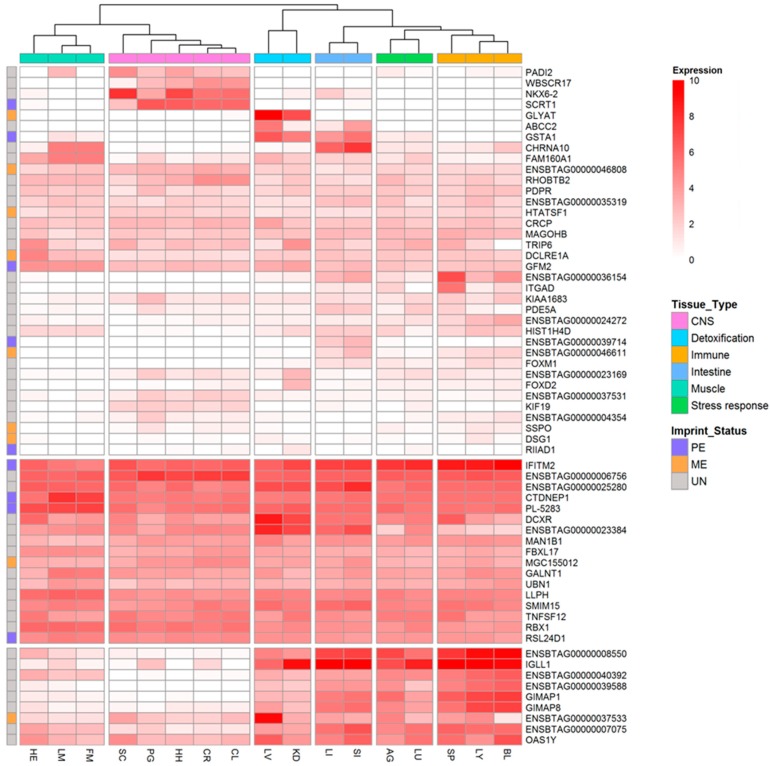
The expressions and imprinting status of monoallelically expressed genes in all tested tissues. Red color scale represents log2(median FPKM + 1). The values above 10 were represented to 10, which means the gene expression is very high in the tissue. FPKM, fragments per Kilobase of exon per million fragments mapped; CNS, central nervous system; PE, paternal expression; ME, maternal expression; UN, unknown.

We investigated the expression patterns of 62 MAE genes. The median value from the three offspring’s FPKMs was used as a parameter reflecting the expression of each gene in certain tissues to avoid limitations derived from an outlier. The log_2_(median FPKM + 1) values were used in heatmap to generate expression clusters (Figure 2).

As expected, there were clear differences in expression features between tissues, and the tissues were classified into six clusters: muscle (cardiac muscle, HE; skeletal muscle, LM and FM), central nervous system (CNS; SC, PG, HH, CR, and CL), detoxification system (LI and KD), intestine (LI and SI), stress response system (AG and LU), and immune system (SP, LY, and BL). In addition, MAE genes were also clustered based on tissue-specific expression. Seventeen genes, including *MGC155012* (maternally expressed), *IFITM2* (paternally expressed), *CTDNEP1* (paternally expressed), *PL-5283* (paternally expressed), and *RSL24D1* (paternally expressed) were highly expressed in all tissues. Nine genes, including *IGLL1*, *GIMAP1*, *GIMAP8*, and *OAS1Y* showed relatively high expression in tissues other than muscle, brain, and spinal cord. Furthermore, *PADI2*, *WBSCR17*, *NKX6*-*2*, and *SCRT1* (maternally expressed) exhibited CNS tissue-specific expression. Maternally expressed *GLYAT* was expressed in only liver and kidney, which were classified as detoxification system-related tissues in this study.

### 3.4. Functional Annotation of MAE Genes

In this study, DAVID and PathwayStudio were used to analyze the functions MAE genes. Ensembl gene IDs were used as input for DAVID and *Bos taurus* was selected under the “species” category. Data for gene ontology for GOTERM_BP_FAT was obtained. However, within the gene set, there were no gene ontology (GO) terms or pathways that clustered significantly. Hence, we categorized the gene set according to the similarity of terms in the Pathwaystudio database and obtained results as shown in Table 2. Six genes (*FOXD2*, *FOXM1*, *HTATSF1*, *SCRT1*, *NKX6*-*2*, and *UBN1*) encode transcription factors, which could regulate downstream genes at the transcriptional level. In addition, *FOXD2* and *FOXM1* belong to the forkhead box (FOX) proteins, and FOX deregulation is known to be associated with many diseases, particularly for *FOXM1*, which is an oncogene that is overexpressed in most types of human cancer [18].

### 3.5. Experimental Validation of MAE Gene Using Direct Sequencing

**Figure 3:**
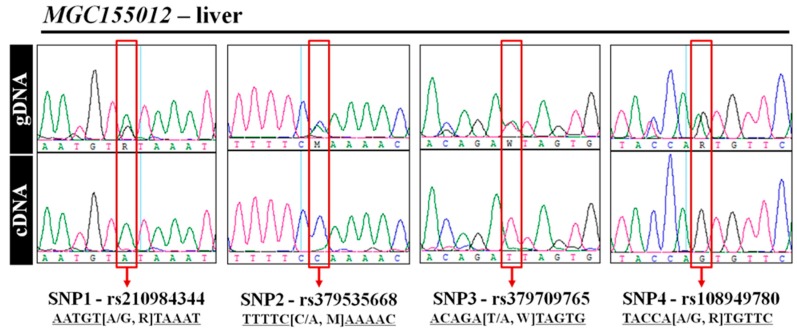
Representative sequence chromatogram for allele-specific expressions of four single nucleotide polymorphisms (SNPs) of *MGC155012* in liver. In each SNP locus, gDNA is heterozygous and cDNA, which was synthesized from RNA, is homozygous.

To validate the results generated from the NGS data, we employed direct sequencing of the offspring’s gDNA and cDNA to identify genomic variations and the expressed alleles, respectively. As shown in Figure 3, all four of the SNPs in *MGC155012* were heterozygous, given the overlap of differently colored peaks at each locus. In contrast to this, in the case of cDNA, only one peak was displayed at each locus. This means that all heterozygous SNPs of *MGC155012* were expressed monoallelically in liver. We obtained the same results for cDNAs in other tissues. In the same way, the presence of MAE of two genes (*Martin3*, *RBX1*, *ENSBTAG00000016502*, and *SERPINB6*) were also confirmed (Figure S4). These results show that MAE patterns were present in adult cattle, and these genes might influence cattle phenotypes. Therefore, further studies for validation of MAE patterns in the reciprocal population and association of MAE genes with economic traits still remain to be studied.

## 4. Discussion

The genomic imprinting, which is a subtype of MAE, is distinct from dominance effects, which are independent of the origin of the alleles, even though the recessive allele is similarly silenced [19]. More than 100 imprinted genes have been discovered so far that are involved not only in embryonic development and growth but also in postnatal behavior and metabolism [20,21]. Therefore, in farm animals, imprinted genes are considered as candidate targets for artificial selection for economically important traits. However, a relatively small number of imprinted genes (20 genes in cattle and 22 genes in pigs; http://www.geneimprint.com/site/genes-by-species; 26 July 2019) have been reported in farm animals compared with humans and mice.

Hanwoo is an indigenous meat-type breed in Korea, and Hanwoo breeding strategies have concentrated on using a Korean proven bull (KPN) program for artificial insemination since the 1960s [22]. However, the cows make up half of the genetic backbone of their offspring. So, currently, a need for cow breeding programs to enhance genetic gain in Hanwoo has been realized. Progeny tests for cows are more expensive and time-consuming compared with progeny tests for bulls. Therefore, genotype-based selection targeting the parent-specific expressed genes could be more effective for cow breeding. This is one of the initial studies to identify ASE patterns in Hanwoo and we focused on identifying the MAE status in adult animals because it is believed that postnatal MAE contribute better to phenotypic variations compared with prenatal MAE.

In this study, a total of 62 genes were found to have MAE pattern in at least one tissue. Among them, 31 genes were shared with the MAE genes previously reported in humans [23]. In particular, 10 genes (*PADI2*, *HIST1H4D*, *GIMAP1*, *PDE5A*, *RHOBTB2*, *IFITM2*, *IGLL1*, *DCXR*, *TNFSF12,* and *TRIP6*) had MAE in both humans and mice [23]. Several genes (*RBX1*, *FOXM1*, *MAN1B1*, *PDPR*, *CTDNEP1,* and *UBN1*) were biallelically expressed in humans and/or mice. These data suggest that MAE patterns vary depending on species which is in concordance with previous research [24,25]. There are other similar cases of species-specific MAE in cattle. The bovine genes such as *Nesp55*, *NNAT*, *NAP1L5*, and *H19* show MAE similar humans and mice, whereas *SLC38A4* has biallelic expression, unlike its counterpart in humans and mice [26,27]. Among these genes, *Nesp55* and *NNAT* showed MAE in both the fetus and adult tissues [27]. Chen et al. (2016) identified 53 MAE genes in bovine conceptus from *Bos indicus* × *Bos Taurus* F_1_ cross [8]. They also reported the MAE status of *Nesp55* and validated the differentially methylated region (DMR) in the promoter regions that was associated with the maternal expression. In the current study, there were no SNPs in these genes that were heterozygote in all offspring, so it was not possible to test MAE status for these genes. Here, we identified 31 MAE genes which were not listed in bovine conceptus. Recently, it has been reported that MAE pattern changes with embryonic developmental stages by single-cell RNA sequencing [28].

The MAE genes found in this study showed tissue-specific expression patterns. Similar results with higher expression of *ABCC2* in the liver than that in the small intestine or kidney have been observed in other studies [29]. The transcription factor *NKX6-2*, which is specifically expressed in the brain, was also previously considered to be a MAE gene in humans [19,20]. Recently, it has also been reported that *FOXM1* induces methylation signatures, resulting in epigenetic regulation [30]. Regarding other FOX family genes, *FOXG1* and *FOXF1* were also predicted to be MAE genes [31]. *PDE5A*, *MAN1B1*, *WBSCR17*, and *GALNT1* are known to be associated with translational regulation. Besides these examples, some of the other MAE genes found in this study are related to metabolic processes, immune responses, and the cell cycle.

## 5. Conclusions

In this study, parental origins of the expressed allele for 18 bovine MAE genes were identified. Taken together, this study supports the time and tissue-specific MAE patterns in cattle. These findings could form the basis for further studies to understand the epigenetic mechanisms and phenotypic variation behind MAE patterns in cattle.

## Figures and Tables

**Table 1:** Summary of monoallelically expressed genes in tested tissues.

Tissues	Tested SNPs ^1^	Tested Genes	MAE Genes ^2^				MAE%
			PE	ME	UN	Total	
Pituitary gland	679	467	6	3	15	24	5.14%
Hypothalamus	823	561	5	3	19	27	4.81%
Spinal cord	467	327	4	2	12	18	5.50%
Lymph node	1104	654	4	5	23	32	4.89%
Spleen	1131	676	4	3	23	30	4.44%
Liver	852	515	6	5	15	26	5.05%
Large intestine	1000	594	6	2	20	28	4.71%
Small intestine	1042	613	7	3	20	30	4.89%
Heart	593	388	4	2	17	23	5.93%
Loin muscle	508	340	4	2	13	19	5.59%
Femoral muscle	593	397	4	2	15	21	5.29%
Kidney	946	589	7	3	22	32	5.43%
Adrenal gland	853	531	4	3	17	24	4.52%
Blood	823	511	4	3	17	24	4.70%
Lung	1047	644	5	2	20	27	4.19%
Cerebrum	988	666	5	4	17	26	3.90%
Cerebellum	1043	699	6	4	18	28	4.01%

^1^ Number of heterozygous SNPs available in all offspring in each tissue; ^2^ Monoallelic expression status: PE, paternal expression; ME, maternal expression; UN, unknown.

**Table 2:** Functional annotations of MAE genes based on PathwayStudio database.

Gene Symbol	Gene Description	BTA	Status	Cell Localization	Class
**Transcriptional regulation**					
*FOXD2*	*forkhead box D2*	3	UN	Nucleus	TF
*FOXM1*	*forkhead box M1*	5	UN	Nucleus	TF
*HTATSF1*	*HIV-1 Tat specific factor 1*	12	ME	Nucleus	TF
*SCRT1*	*scratch family zinc finger 1*	14	PE	Nucleus	TF
*NKX6-2*	*NK6 homeobox 2*	18	UN	Nucleus	TF
*TRIP6*	*thyroid hormone receptor interactor 6*	25	UN	Cytoplasm	-
*UBN1*	*ubinuclein 1*	25	UN	Nucleus	TF
**Translational Regulation**					
*PDE5A*	*phosphodiesterase 5A, cGMP-specific*	6	UN	.	-
*MAN1B1*	*mannosidase, alpha, class 1B, member 1*	11	UN	ER membrane	-
*WBSCR17*	*Williams-Beuren syndrome chromosome region 17*	17	UN	Golgi apparatus	-
*GALNT1*	*polypeptide N-acetylgalactosaminyltransferase 1*	24	UN	Golgi apparatus	-
**Metabolic Process**					
*GLYAT*	*glycine-N-acyltransferase*	15	ME	Mitochondrion	-
*PDPR*	*pyruvate dehydrogenase phosphatase regulatory subunit*	18	UN	Mitochondrion	-
*KIF19*	*kinesin family member 19*	19	UN	Cytoplasm	-
*DCXR*	*dicarbonyl/L-xylulose reductase*	19	UN	Membrane	-
*GSTA1*	*glutathione S-transferase alpha 1*	23	PE	Cytoplasm	-
*ABCC2*	*ATP-binding cassette, sub-family C (CFTR/MRP), member 2*	26	UN	Apical cell membrane	Transporter
**Immune Response**					
*TNFSF12*	*tumor necrosis factor (ligand) superfamily, member 12*	19	UN	Cell membrane	Ligand
*CRCP*	*CGRP receptor component*	25	UN	Nucleus	Receptor
*ITGAD*	*integrin, alpha D*	25	UN	Membrane	Receptor
**Cell Cycle**					
*HIST1H4D*	*histone cluster 1, H4d*	3	UN	Nucleus	-
*SSPO*	*SCO-spondin*	4	ME	Extracellular space	-
*CHRNA10*	*cholinergic receptor, nicotinic,* *alpha 10 (neuronal)*	15	UN	Cell junction	-
*CTDNEP1*	*CTD nuclear envelope phosphatase 1*	19	PE	ER membrane	Protein phosphatase
*DSG1*	*desmoglein 1*	24	ME	Cell membrane	-
*DCLRE1A*	*DNA cross-link repair 1A*	26	ME	Nucleus	-

Abbreviation: PE, paternal expression; ME, maternal expression; UN, unknown; ER, endoplasmic reticulum; TF, transcription factor.

## Supplementary Materials

The following are available online at https://www.mdpi.com/2076-2615/9/10/727/s1, Figure S1: The illustration of data analysis steps for the identification of MAE genes, Figure S2: The numbers of total SNPs and exonic SNPs identified in Hanwoo, Figure S3: The number of exonic SNPs in each offspring and the number of informative SNPs, Figure S4: Sequence chromatogram for allele-specific expressions of (a) MATR3 gene (b) RBX1 gene (c) ENSBTAG00000016502 gene and (d) SERPINB6 gene, Table S1: Primers sequence for validation of imprinted genes using direct sequencing, Table S2: Summary statistics for resequencing data, Table S3: Summary statistics for RNA-sequencing data.
