# Supplementary material for: Genome-Wide Analysis of Allele-Specific Expression Patterns in Seventeen Tissues of Korean Cattle (Hanwoo)
Source: Animals (Basel). 2019 Sep 26;9(10):727. doi: 10.3390/ani9100727 (PMC6826869; doi:10.3390/ani9100727)
Supplement: Supplementary file 1 [file animals-09-00727-s001.zip › Table S1 - Revised.docx]

**Table S1.** Primers sequence for validation of imprinted genes using direct sequencing.

| Name | Sequence (5´ to 3´) | Size | Target gene | Target SNPs |
| --- | --- | --- | --- | --- |
| P1-F  P1-R | CTGATGATTCCAATGGGCTGG  GGCACAGAAAAGGAAGTGAAAAAC | 872 bp | *MGC155012*  (*ENSBTAG00000003941*) | rs210984344  rs379535668  rs379709765  rs108949780 |
| P2-F  P2-R | TGAAGCATTTTTGGTTTTGTTTCA  ACTTGAATCCACAGATATAAGCAGT | 530 bp | *Martin 3*  (*ENSBTAG00000006756*) | rs136120832  rs110453249  rs109851622 |
| P3-F  P3-R1 (gDNA)  P3-R2 (cDNA) | TTATCTTGGCTGGCAGTGGA  CCCACCCATTCCTGCCTTATT  CAGAAGACAGCTGGATTCGC | 565 bp  379 bp | *RBX1*  (*ENSBTAG00000012081*) | rs132828050  rs137134202  rs210087802 |
| P4-F  P4-R | GATGGCCGTCCCTACACAAA  GCAGGAAGAAAGAGGGTTTTTATTT | 501 bp | *ENSBTAG00000016502* | rs209432312 |
| P5-F  P5-R1 (gDNA)  P5-R2 (cDNA) | AATTAGAGACTTGCTCCCTGC  GAGGCACACGTTCCTTGAGA  GGTCTTGCTGTGCTGAATGAA | 249 bp  668 bp | *SERPINB6*  (*ENSBTAG00000025280*) | rs207891256 |
