# Supplementary figures and images for: Genome-Wide Analysis of Allele-Specific Expression Patterns in Seventeen Tissues of Korean Cattle (Hanwoo)
Source: Animals (Basel). 2019 Sep 26;9(10):727. doi: 10.3390/ani9100727 (PMC6826869; doi:10.3390/ani9100727)

## Slide 1
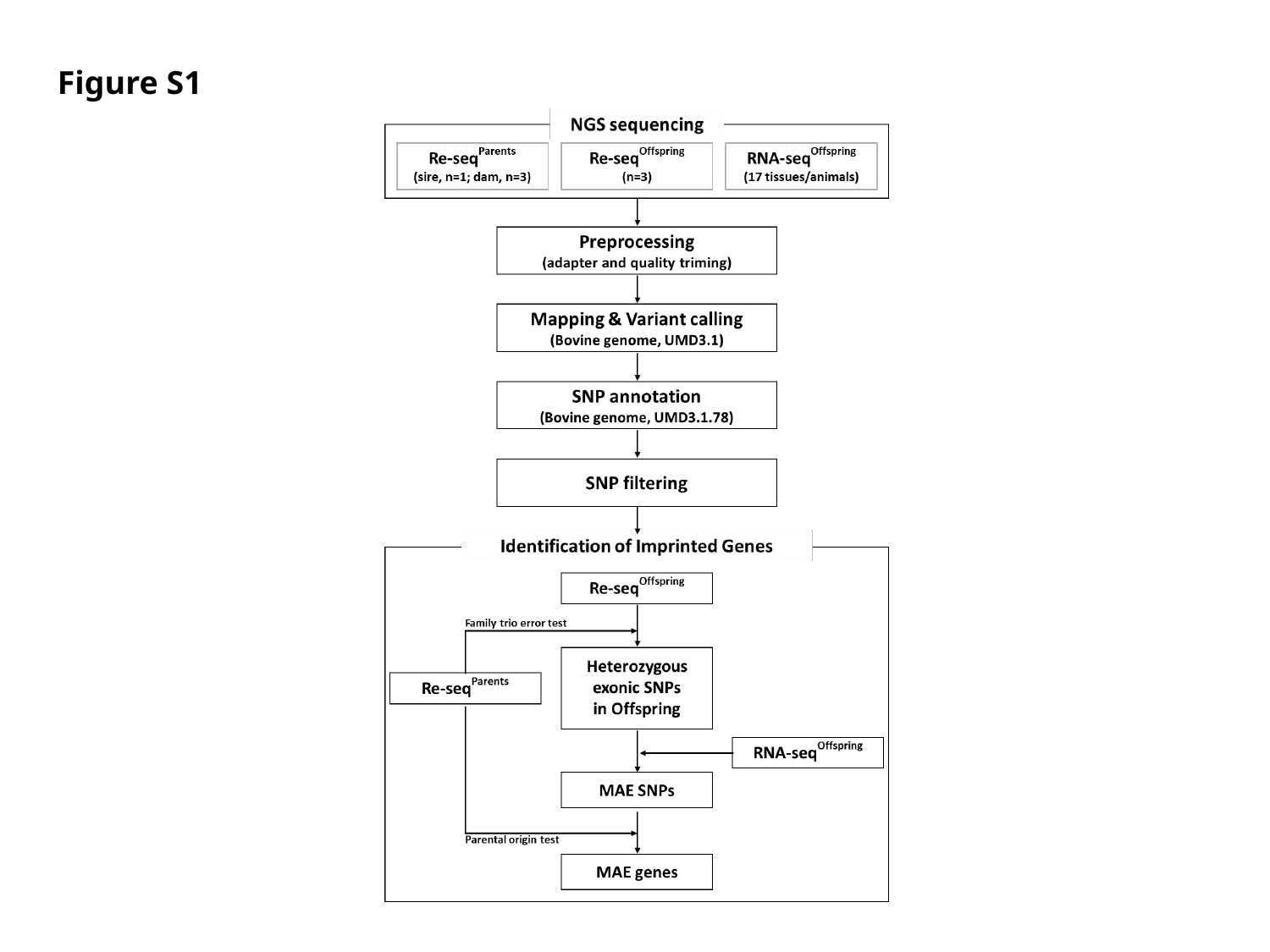

Figure S1

## Slide 2
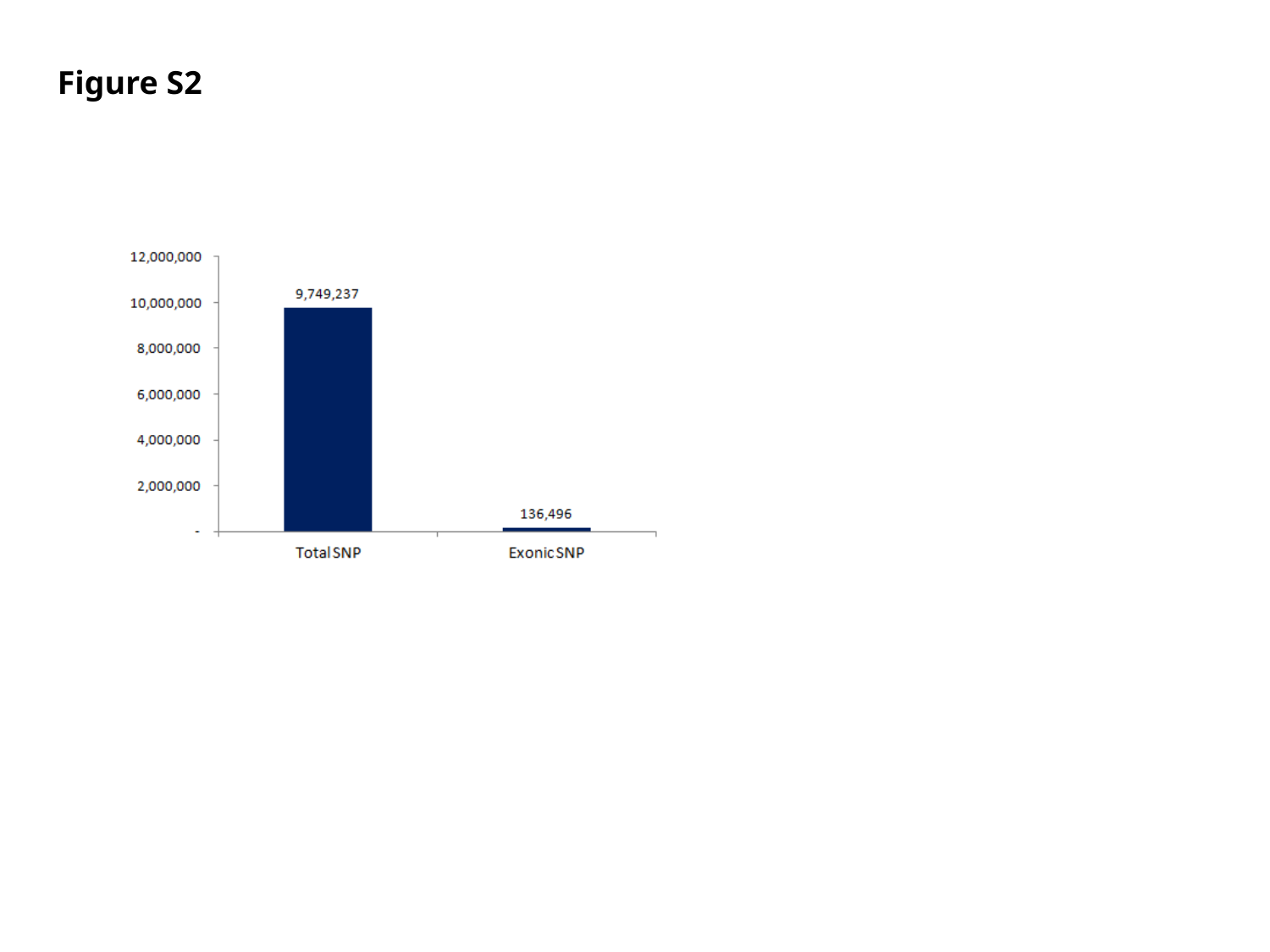

Figure S2

## Slide 3
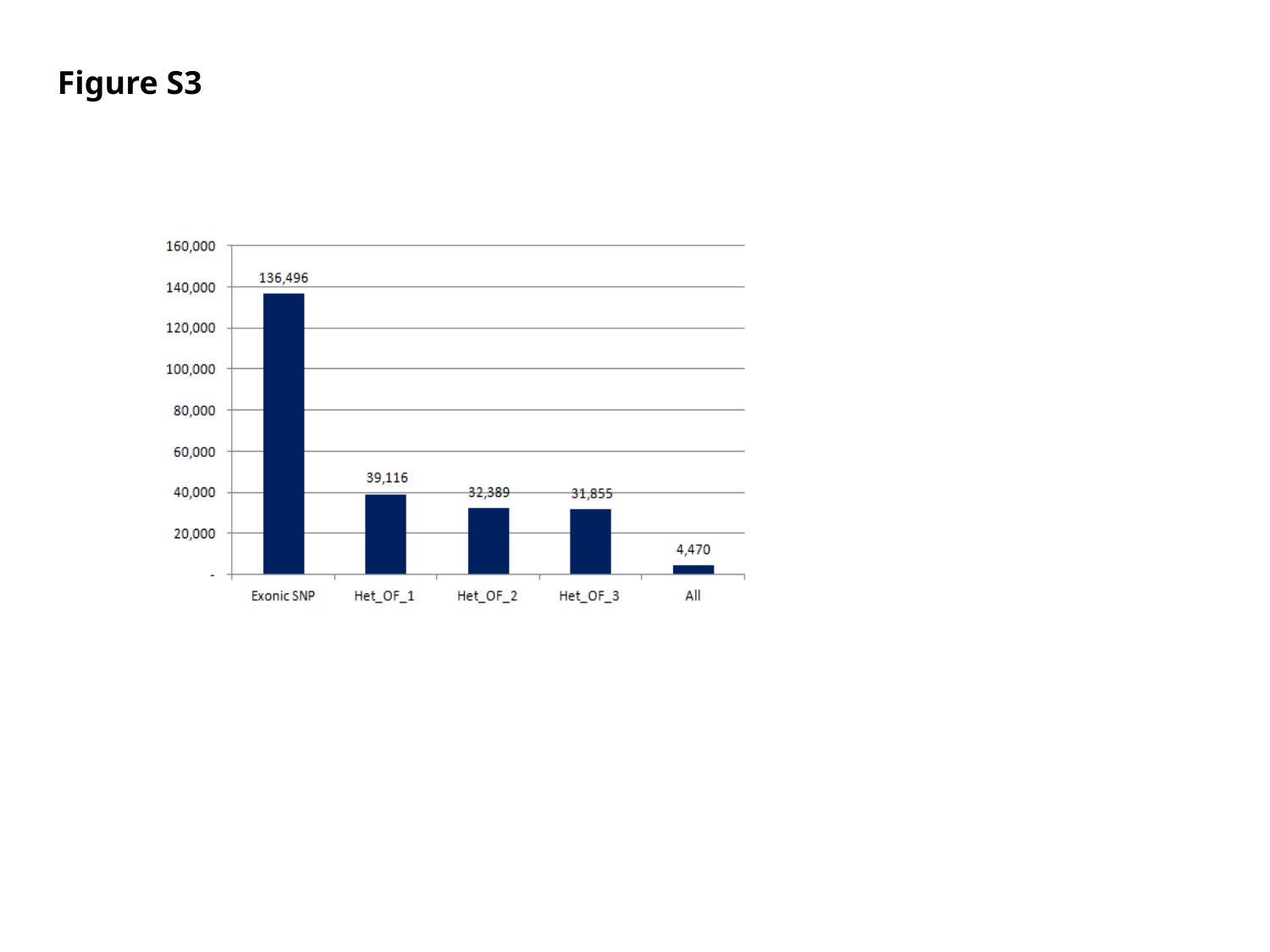

Figure S3

## Slide 4
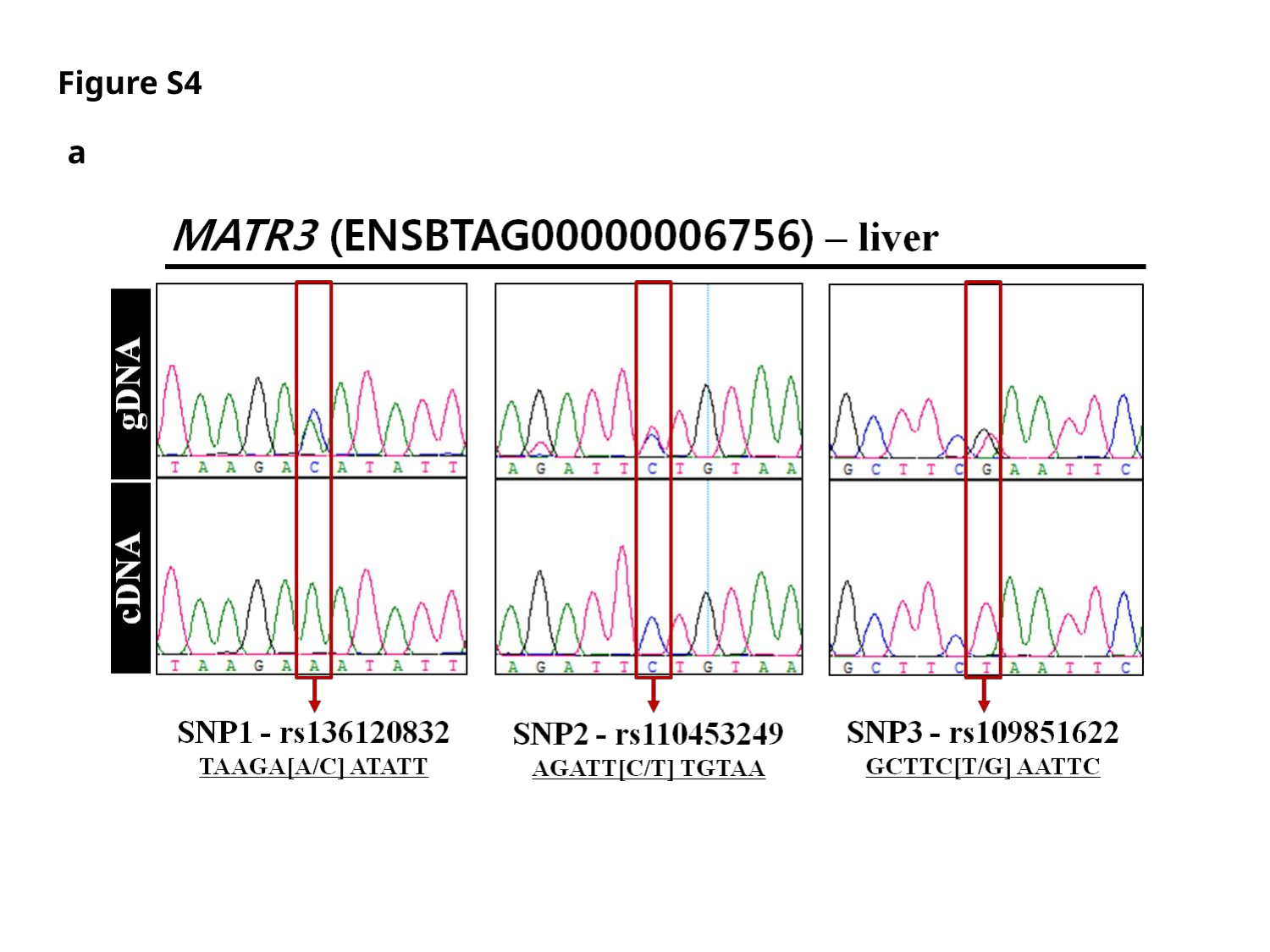

Figure S4
a

## Slide 5
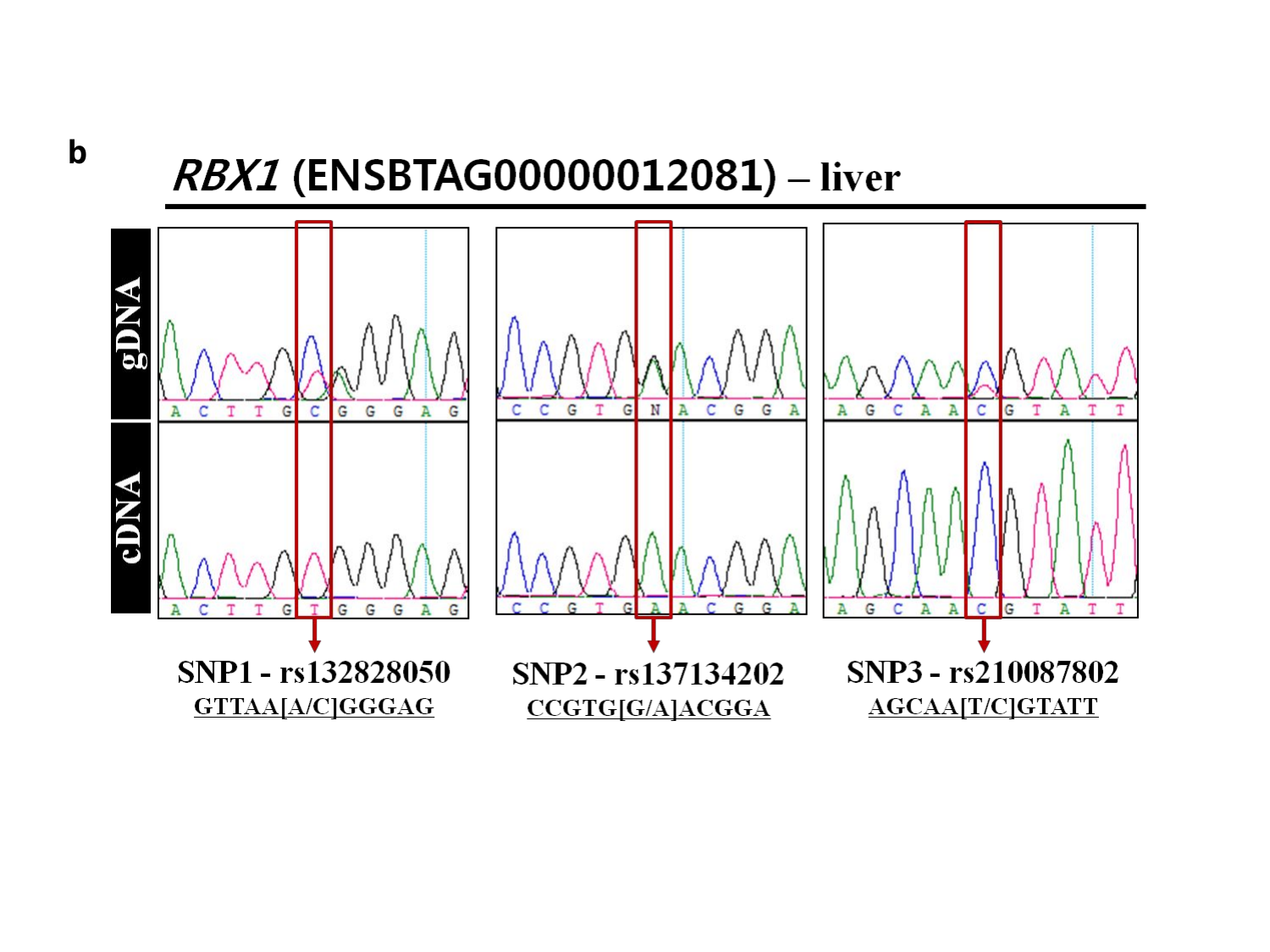

b

## Slide 6
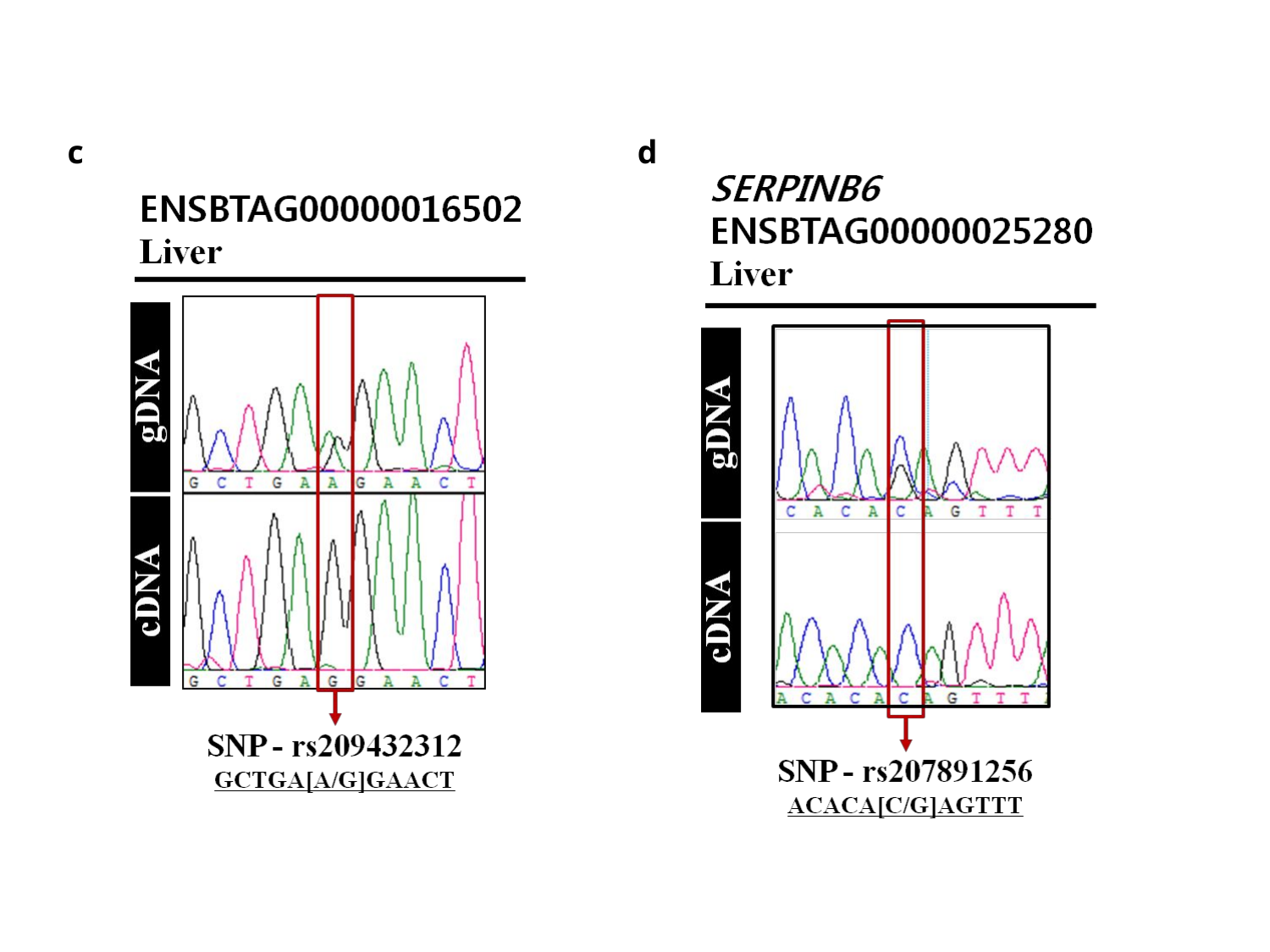

c
d

Supplement: Supplementary file 1 [file animals-09-00727-s001.zip › Figure S1-S3 - Revised.pptx]
